# Successful conversion surgery for stage IV gastric cancer with liver metastases after second-line chemotherapy with ramucirumab and paclitaxel: a case report

**DOI:** 10.1186/s40792-022-01412-x

**Published:** 2022-04-01

**Authors:** Kosuke Fukuda, Takaaki Arigami, Koki Tokuda, Shigehiro Yanagita, Daisuke Matsushita, Yota Kawasaki, Satoshi Iino, Ken Sasaki, Akihiro Nakajo, Mari Kirishima, Akihide Tanimoto, Hitoshi Tsubouchi, Hiroshi Kurahara, Takao Ohtsuka

**Affiliations:** 1 Department of Digestive Surgery, Breast and Thyroid Surgery, Kagoshima University Graduate School of Medical and Dental Sciences, 8-35-1 Sakuragaoka, Kagoshima, 890-8520 Japan; 2 Department of Onco-Biological Surgery, Kagoshima University Graduate School of Medical and Dental Sciences, 8-35-1 Sakuragaoka, Kagoshima, 890-8520 Japan; 3 Department of Gastrointestinal Surgery, Kobayashi City Hospital, Miyazaki, Japan; 4 Department of Pathology, Kagoshima University Graduate School of Medical and Dental Sciences, Kagoshima, Japan

**Keywords:** Gastric cancer, Second-line chemotherapy, Conversion surgery

## Abstract

**Background:**

In recent years, conversion surgery after chemotherapy has been considered a promising strategy for improving the prognosis of patients with stage IV gastric cancer. However, there are few reports on conversion gastrectomy after second-line chemotherapy. Here, we report a case of long-term survival of a patient with liver metastases from gastric cancer who underwent conversion surgery after second-line chemotherapy with ramucirumab and paclitaxel.

**Case presentation:**

A 77-year-old man complaining of weight loss was diagnosed with human epidermal growth factor receptor 2-positive gastric cancer with multiple liver metastases. Although the patient initially received trastuzumab-based chemotherapy, it was discontinued, because he experienced trastuzumab-induced infusion reactions. Thereafter, he was treated with six courses of S-1 plus cisplatin and six courses of ramucirumab plus paclitaxel as the first- and second-line regimens, respectively. The primary tumor and liver metastases remarkably shrank, and the reduction rate of the measurable metastatic liver lesions was 81.1%. According to the Response Evaluation Criteria in Solid Tumors, the patient responded partially. Therefore, he underwent total gastrectomy with D2 lymphadenectomy and partial hepatectomy of segments 3 and 4. Pathological examination revealed tumor invasion into the muscularis propria, a grade 1a histological response, and no lymph node metastases. No viable cancer cells were identified in the specimens resected from liver segments 3 and 4. Accordingly, the patient was pathologically diagnosed with stage IB (ypT2N0M0). Postoperatively, the patient received adjuvant chemotherapy with S-1 for 6 months, and he survived without recurrence for 42 months after conversion surgery.

**Conclusions:**

Conversion surgery might be clinically useful for improving survival in certain patients with gastric cancer, including those who previously received second-line chemotherapy.

## Background

Gastric cancer is the fourth most common cancer worldwide and the second leading cause of cancer-related death [1]. Systemic chemotherapy is currently recommended for patients with stage IV gastric cancer [2]. Notably, the Japanese Gastric Cancer Treatment Guidelines categorize each recommended regimen as first-, second-, or third-line chemotherapy [2]. The RAINBOW trial, a double-blind, randomized phase 3 study of patients with advanced gastric or gastroesophageal junction adenocarcinoma who received first-line chemotherapy, demonstrated that the combination of ramucirumab (RAM) and paclitaxel (PTX) had better clinical utility for increasing overall survival (OS) than placebo plus PTX [3]. Consequently, the Japanese Gastric Cancer Treatment Guidelines recommend RAM plus PTX for second-line chemotherapy in patients with unresectable advanced or recurrent gastric cancer [2].

In recent years, conversion surgery after chemotherapy has been considered a promising strategy for improving the prognosis of patients with stage IV gastric cancer [4, 5]. Many investigators have demonstrated the prognostic impact of conversion surgery after chemotherapy for stage IV gastric cancer [6–8]. However, in most reports, conversion surgery was performed in responders after first-line chemotherapy [9–11], and there are few reports of conversion surgery in responders after second-line chemotherapy with RAM and PTX. Herein, we report a case of long-term survival of a patient with gastric cancer and liver metastases who underwent conversion surgery due to partial response to systemic second-line treatment with RAM plus PTX.

## Case presentation

**Fig. 1:**
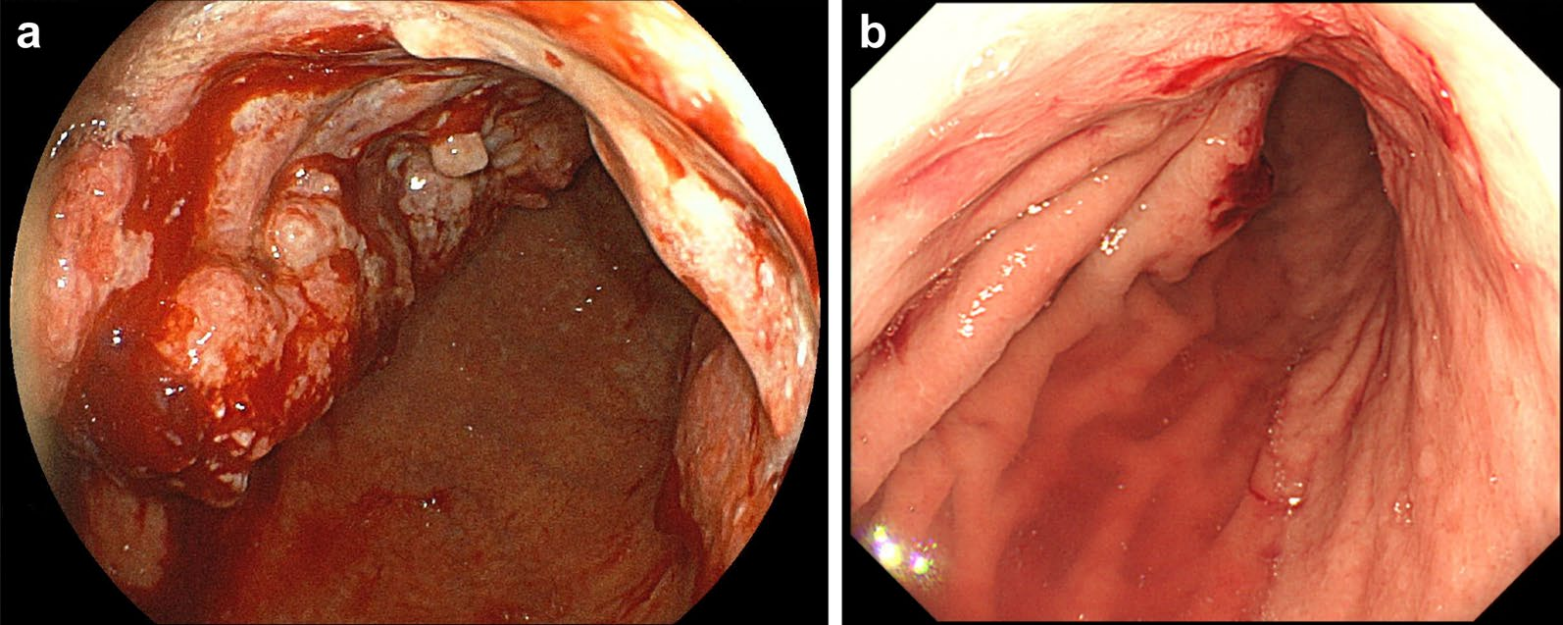
Esophagogastroduodenoscopy image. **a** Before chemotherapy. A type 2 tumor that extends from the upper to middle third of the stomach is seen. **b** Size of the primary tumor has remarkably reduced after six courses of ramucirumab plus paclitaxel

**Fig. 2:**
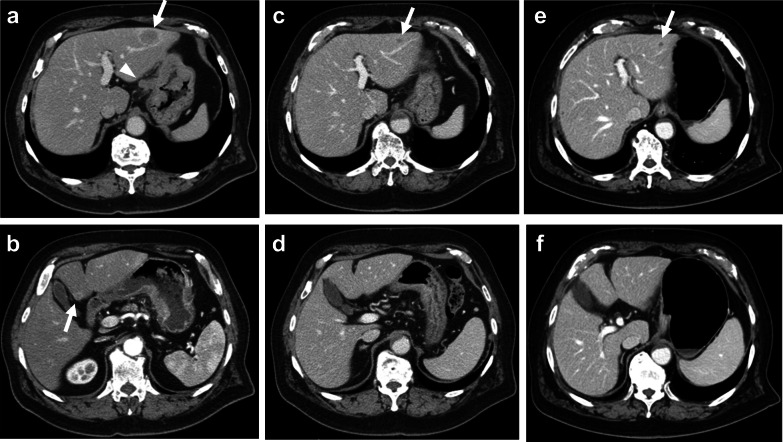
Enhanced computed tomography. **a**, **b** Before chemotherapy. Metastases at the station 3 lymph nodes (arrowhead) and segments 3 and 4 of the liver (arrows) are seen. **c**, **d** After three courses of S-1 plus cisplatin, the lymph node and liver metastases (arrow) remarkably reduced in size. **e**, **f** After six courses of ramucirumab plus paclitaxel, the tumor shrinkage of the lymph node and liver metastases (arrow) was maintained

**Fig. 3:**
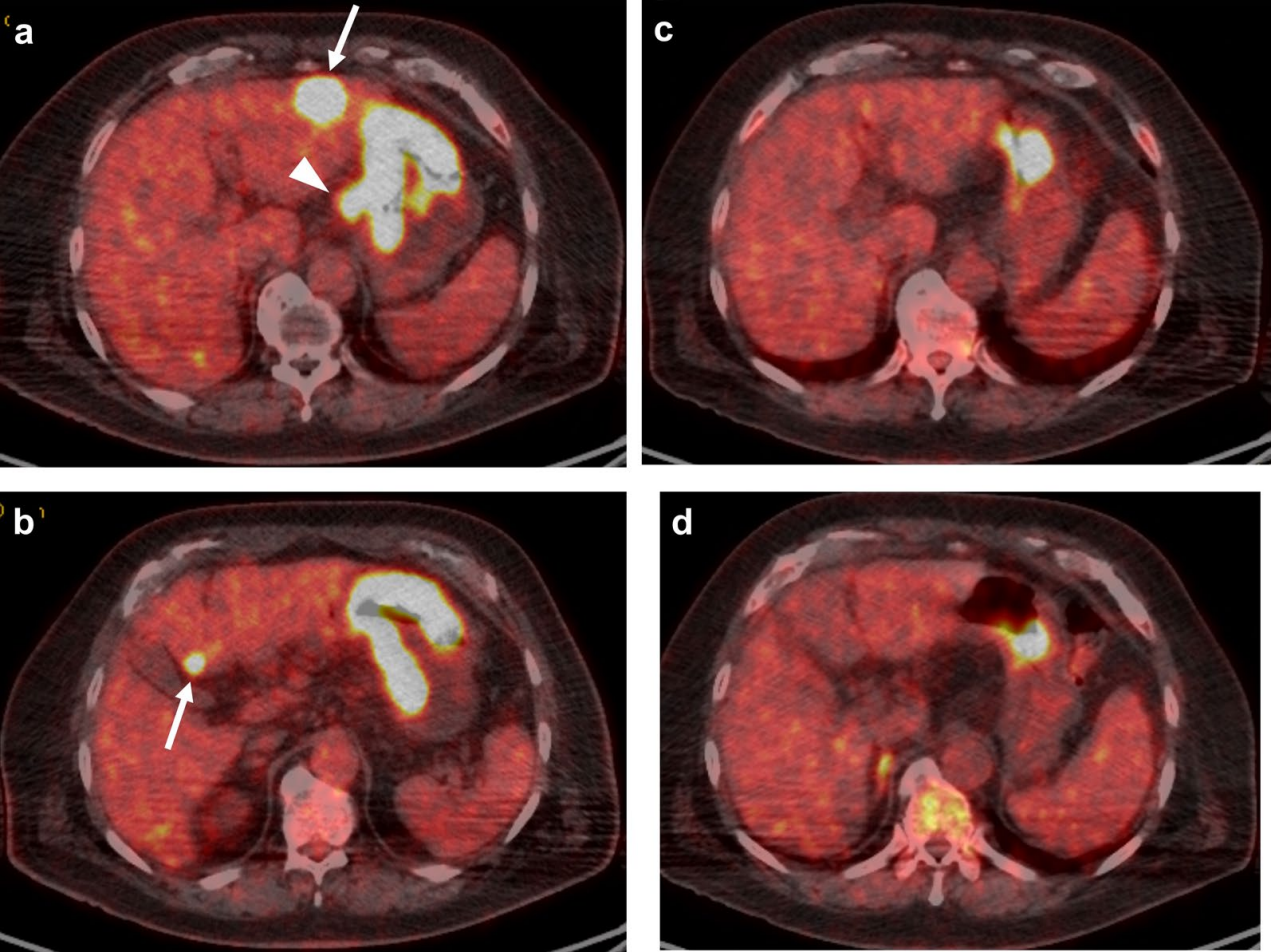
Positron emission tomography–computed tomography. **a**, **b** Before chemotherapy. Abnormal uptake in the primary tumor, enlarged station 3 lymph nodes (arrowhead), and tumors in the segments 3 and 4 of the liver (arrows) are seen. **c**, **d** After six courses of ramucirumab plus paclitaxel, the abnormal uptake in the lymph node and liver metastases disappeared

**Fig. 4:**
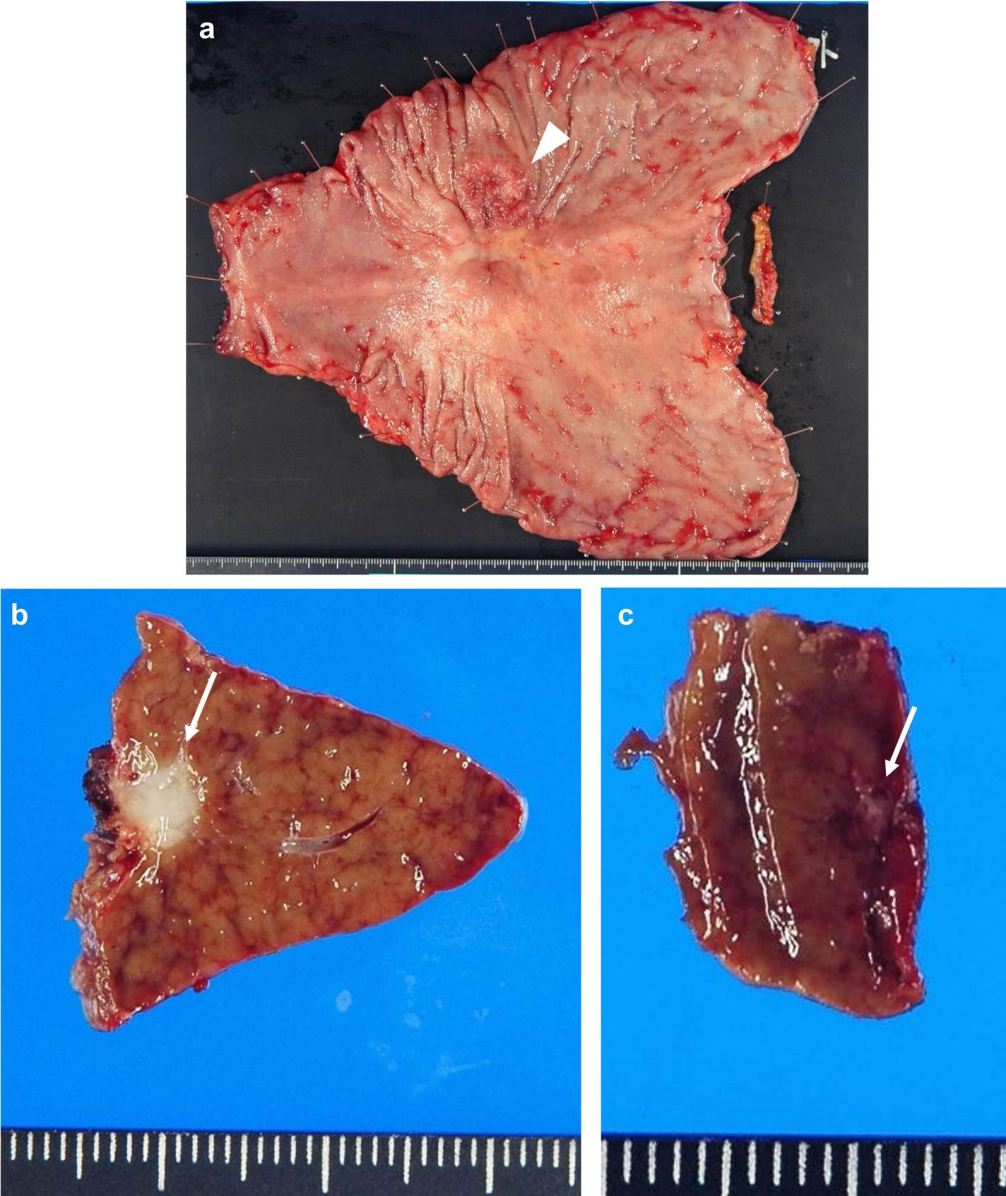
Macroscopic findings of the resected stomach (**a**) and liver (**b**, **c**). A type 5 primary tumor (arrowhead) and white tumor scar at liver segments 3 and 4 (arrows) are seen

**Fig. 5:**
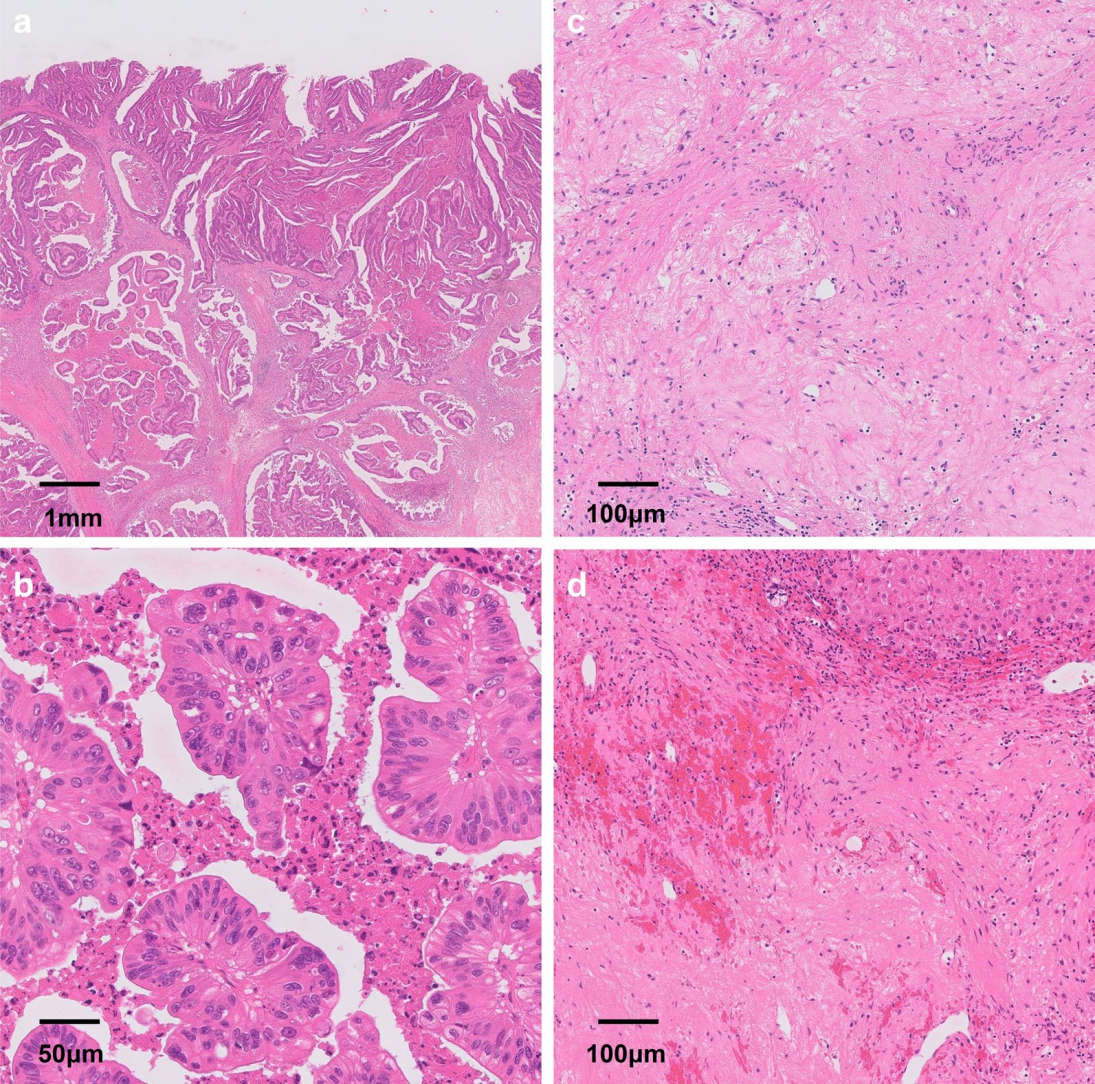
Pathological findings. **a** Primary tumor of the resected stomach is composed of well-differentiated adenocarcinoma invading to the muscularis propria (ypT2). **b** The majority of tumor cells are viable (grade 1a). No viable tumor cells are seen in the specimens resected from liver segments 3 (**c**) and 4 (**d**)

A 77-year-old man visited a local hospital with a chief complaint of weight loss. His serum carcinoembryonic antigen (CEA) level was 371.5 ng/mL. Esophagogastroduodenoscopy revealed a half-circumferential type 2 tumor extending from the upper to the middle third of the stomach (Fig. 1a). Pathological examination of the biopsied specimens revealed well-differentiated adenocarcinoma. Immunohistochemistry results showed that the biopsied tumors had a human epidermal growth factor receptor 2 (HER2) score of 3 + . Enhanced computed tomography revealed gastric wall thickening at the tumor site, enlarged station 3 lymph nodes along the lesser curvature, and liver metastases in segment 3 (S3) and segment 4 (S4) (Fig. 2a, b). Positron emission tomography–computed tomography (PET–CT) revealed abnormal uptake in the primary tumor, enlarged station 3 lymph nodes, and tumors in the liver at S3 and S4 (Fig. 3a, b). Consequently, the patient was clinically diagnosed with stage IV HER2-positive gastric cancer (cT4aN3M1), and chemotherapy with capecitabine, cisplatin, and trastuzumab was planned. However, severe infusion reactions occurred during the initial administration of trastuzumab, and this regimen was discontinued. Thereafter, the patient received S-1 plus cisplatin (SP) as first-line chemotherapy. This regimen consisted of a 5-week course of S-1 (100 mg/body/day), administered orally on days 1–21, with cisplatin (60 mg/m^2^), administered intravenously on day 8. After three courses of SP, the lymph node and liver metastases were remarkably reduced in size (Fig. 2c, d). After six courses of SP, the primary tumor size progressed. The serum CEA level decreased to 8.5 ng/mL after three courses of SP, but elevation to 44.1 ng/mL occurred after 6 courses of SP. Since the tumor activity was uncontrolled, second-line chemotherapy with RAM and PTX was initiated. This regimen consisted of a 4-week course of PTX (80 mg/m^2^), administered intravenously on days 1, 8, and 15, and RAM (8 mg/kg), administered intravenously on days 1 and 15. After six courses of RAM plus PTX, the primary tumor size remarkably reduced (Fig. 1b), and the shrinkage of the liver metastases was maintained (Fig. 2e, f). Furthermore, the serum CEA level decreased to 9.4 ng/mL. PET–CT revealed no abnormal uptake within the lymph node and liver metastases and reduced tumor activity at the primary site (Fig. 3c, d). The patient showed a partial response, with an 81.1% reduction according to the Response Evaluation Criteria in Solid Tumors. Therefore, conversion surgery was considered. The patient was referred to our hospital for the surgery. After ruling out peritoneal dissemination and obtaining staging laparoscopy findings of negative peritoneal cytology, the patient underwent total gastrectomy with D2 lymphadenectomy plus partial hepatectomy of S3 and S4. Macroscopically, a type 5 primary tumor and white tumor scar at S3 and S4 were seen (Fig. 4a–c). Pathological examination revealed that the primary tumor invaded the muscularis propria (ypT2), indicating a histological grade of 1a (Fig. 5a, b). No tumor cells were seen in the dissected lymph nodes (ypN0) and specimens resected from S3 and S4 of the liver (Fig. 5c, d). Accordingly, the patient was pathologically diagnosed with stage IB (ypT2N0M0). The patient experienced no surgical complications and was discharged on the tenth postoperative day. He received oral S-1 as an adjuvant chemotherapy. However, it was discontinued after 6 months due to the occurrence of anorexia and malaise. The patient is alive 57 months after the first-line chemotherapy, and there were no signs of disease recurrence at 42 months postoperatively.

## Discussion

We describe a patient with stage IV gastric cancer with multiple liver metastases who successfully underwent gastrectomy and hepatectomy as conversion surgery after second-line chemotherapy with RAM and PTX. Moreover, pathological examination showed that lymph node metastasis and liver metastases disappeared due to chemotherapy. Although the RAINBOW trial showed that second-line chemotherapy with RAM and PTX contributes to prognostic improvements, including improvements in tumor response, in patients with unresectable advanced gastric cancer, the clinical significance of conversion surgery in responders to chemotherapy with RAM and PTX remains unclear [3]. We searched for literature on conversion surgery after second-line chemotherapy for gastric cancer in the PubMed database using the terms "gastric cancer" and "conversion surgery" and excluded articles published only in abstract form. To our knowledge, this is the third report on conversion surgery after second-line treatment with RAM-based chemotherapy in patients with advanced gastric cancer [12, 13].

Despite remarkable advances in chemotherapy, the prognosis of patients with stage IV gastric cancer remains poor. The liver is the most common site of distant metastasis from gastric cancer, and the incidence of liver metastasis is reported to be 48% in patients with metastatic gastric cancer [14]. Surgical resection is generally recommended for patients with colorectal cancer who have liver metastases that are amenable to R0 resection [15]. In contrast, the clinical impact of surgical resection for liver metastases from gastric cancer remains controversial. However, recent studies have demonstrated that hepatectomy may have clinical significance for improving prognosis in patients with liver-limited metastasis from gastric cancer [7, 8]. Oki et al. reported 3- and 5-year postoperative OS rates of 51.4% and 42.1%, respectively, in 94 patients with liver-limited metastasis who underwent surgery [7]. They concluded that patients with a single liver metastasis from gastric cancer with a nodal status of < N2 might be good candidates for liver resection [7]. Similarly, in their study of 44 patients with liver metastasis from gastric cancer with or without other metastases who were treated with chemotherapy, Arigami et al. found that patients with no peritoneal dissemination who responded to chemotherapy were good candidates for conversion surgery [8]. Despite weak evidence, the Japanese Gastric Cancer Treatment Guidelines also recommend surgical resection in patients with oligometastases who have no noncurative factors [2]. Our patient presented with liver-limited metastasis in the form of two metastatic liver nodules. Furthermore, staging laparoscopy revealed no peritoneal dissemination. Therefore, our patient may be a good candidate for conversion surgery.

Bevacizumab (BV) and RAM are anti-angiogenic agents associated with vascular endothelial growth factor. Therefore, these agents have been clinically suspected to increase postoperative morbidity after surgery by wound healing disorders. Kesmodel et al. recommended waiting at least 6 weeks from discontinuation of BV to surgery in patients undergoing hepatic surgery after neoadjuvant BV for colorectal cancer liver metastases [16]. On the other hand, there is actually little evidence available to judge the safety and optimal timing of surgery in patients with malignancies, including gastric cancer, who receive RAM-based chemotherapy. Consequently, further work is warranted to assess them in patients with stage IV gastric cancer who undergo conversion surgery after RAM-based chemotherapy.

The patient received SP as first-line chemotherapy. Although the primary tumor progressed after six courses of SP, the shrinkage of lymph node metastasis and liver metastases was maintained. These findings suggest that at least first-line chemotherapy including S-1 had a favorable response to these metastases. Moreover, the patient has a high recurrent potential of liver metastases. Accordingly, he received oral S-1 as an adjuvant chemotherapy.

Yoshida et al. proposed a new system for classifying stage IV gastric cancer based on tumor properties and described clinical indications for conversion surgery after chemotherapy [4]. They classified patients with stage IV gastric cancer into four categories, with category 2 denoting patients with marginally resectable metastasis [4]. Based on this classification, conversion surgery is recommended in responders classified as category 2, indicating a complete response or partial response to intensive chemotherapy [4]. According to this classification system, our patient was categorized as category 2. Accordingly, conversion surgery may be a therapeutic strategy for improving prognosis in this patient. High response rates and improvements in prognosis are expected with conversion surgery even after second-line and third-line chemotherapy. However, few studies have investigated the clinical impact of conversion surgery after second- and third-line chemotherapy. Consequently, further studies are required to assess the clinical indication and prognostic significance of conversion surgery in patients who respond to second- and third-line chemotherapy.

## Conclusions

We present the case of a patient with stage IV gastric cancer who achieved R0 resection and long-term survival with gastrectomy and hepatectomy after second-line chemotherapy. Our findings suggest that conversion surgery might be clinically useful for improving survival in certain patients, including those who previously received second-line chemotherapy.

## Data Availability

The data sets generated during this study are available from the corresponding author upon reasonable request.

## Abbreviations

BV: Bevacizumab
CEA: Carcinoembryonic antigen
HER2: Human epidermal growth factor receptor 2
OS: Overall survival
PET–CT: Positron emission tomography–computed tomography
PTX: Paclitaxel
RAM: Ramucirumab
S3: Segment 3
S4: Segment 4
SP: S-1 plus cisplatin
